# Aptamers and aptamer-drug conjugates as synthetic immune modulators for cancer immunotherapy

**DOI:** 10.3389/fimmu.2026.1818316

**Published:** 2026-04-23

**Authors:** Fareeha Arshad, Raja Chinnappan, Tanveer Ahmad Mir, Zara Ahmed, Itika Arora, Mohammed Imran Khan, Ahmed Yaqinuddin

**Affiliations:** 1 College of Medicine, Alfaisal University, Riyadh, Saudi Arabia; 2 Tissue/Organ Bioengineering and BioMEMS Lab, Organ Transplant Centre of Excellence, King Faisal Specialist Hospital and Research Centre, Riyadh, Saudi Arabia; 3 King Faisal Specialist Hospital and Research Centre, Jeddah, Saudi Arabia; 4 Biotechnology Centre, Khalifa University, Abu Dhabi, United Arab Emirates

**Keywords:** aptamer-drug conjugates, aptamers, cancer, immunotherapy, therapeutics

## Abstract

Cancer immunotherapy has transformed oncology by harnessing the immune system to recognize and eliminate malignant cells. However, currently available options, including immune checkpoint inhibitors and cellular therapies, remain limited due to immune-related toxicities, high costs, off-target effects, and variable patient responses. These challenges highlight the need for alternative, synthetic immune-modulating strategies with improved precision, safety, and scalability. Aptamers have recently emerged as promising synthetic immune modulators. Owing to their chemical synthesis, small size, low immunogenicity, and extensive chemical tunability, aptamers offer distinct advantages over protein-based biologics, including enhanced tissue penetration, batch-to-batch consistency, and flexible pharmacokinetic optimization. Beyond their established diagnostic applications, aptamers are being applied as therapeutic agents capable of modulating immune checkpoints, cytokine signaling, and immune cell recruitment within the tumor microenvironment. Aptamer-drug conjugates (ApDCs) also represent a powerful extension of this technology, enabling targeted delivery of cytotoxic or immunostimulatory payloads to tumors while minimizing systemic toxicity. Through this review, we aim to provide a comprehensive overview of aptamer-based immunotherapies, encompassing molecular engineering strategies, SELEX optimization, and structural design principles that underpin target specificity and functional activity. We further examine preclinical and emerging clinical progress, translational challenges related to formulation, pharmacokinetics, and regulatory considerations, and the evolving role of ApDCs in cancer treatment. Finally, we discuss future perspectives for aptamer technologies as next-generation synthetic immune modulators, with the potential to complement or surpass conventional immunotherapeutic approaches in precision oncology.

## 1 Introduction

Cancer immunotherapy has revolutionized oncology by using the immune system of the patient to recognize and eliminate tumor cells. Immune checkpoint inhibitors and cellular therapies have shown to provide remarkable clinical responses (1, 2). However, only a handful of such immunotherapies have been approved for clinical applications and have also shown to cause severe immune-related toxicities, including colitis, endocrinopathies, and autoimmunity, and are also very expensive. In addition, the existing cancer immunotherapy options also suffer from other challenges, including off-target effects, limited patient response rates, and logistical hurdles (3). Therefore, these limitations call for alternative or synthetic modalities that can mimic or enhance immune therapies with improved precision and safety.

Recently, aptamers have emerged as a novel class of immune-modulating agents. Aptamers are short single-stranded DNA or RNA oligonucleotides selected *in vitro* to bind targets with very high affinity and specificity (4). Also referred to as chemical antibodies, aptamers fold into unique three-dimensional structures that enable precise molecular recognition of proteins, peptides, and even whole cells. Unlike proteins, aptamers are synthesized chemically, which ensures batch-to-batch consistency and allows a wide range of modifications like 2’-fluoro substitutions, PEGylation, or cholesterol conjugation to enhance their stability and pharmacokinetics (4, 5). Aptamers, being much smaller than antibodies, allow for faster tissue penetration and are generally non-immunogenic (6, 7). These properties make aptamers highly attractive as synthetic immune modulators, as they retain the binding specificity of antibodies and also simultaneously offer versatile chemical tunability and typically lower production cost. Thus, integrating aptamers with immunotherapy can further help improve treatment outcomes by precisely targeting key immune pathways with fewer side effects.

Aptamers have been widely applied across diagnostics and therapeutics. Initially, they were mostly employed as recognition elements in biosensors and diagnostic assays due to their tight target binding (8, 9). However, in the recent decade, researchers have increasingly transitioned aptamers into therapeutic roles and targeted delivery vehicles. Aptamers are being designed as antagonists or agonists of immune receptors to modulate cytokines, block inhibitory receptors on T cells, and also engage other immune checkpoints (10). Aptamers can also be engineered to recruit immune cells to the tumor microenvironment or to deliver immunostimulatory payloads (11). Thus, because of their ability to serve as both targeting ligands and functional inhibitors, aptamers provide a theranostic versatility that bridges diagnostics and therapy. A particularly potent application of aptamer technology is the aptamer-drug conjugates (ApDCs), in which an aptamer is covalently linked to a therapeutic payload and is similar to antibody-drug conjugates but using a nucleic acid carrier (12). The aptamer moiety guides the conjugate to the tumor by recognizing a cell-surface marker, and then the attached drug is released inside or near the cancer cell. ApDCs can thus markedly concentrate cytotoxic agents in tumors and spare healthy tissues, thereby enhancing anti-tumor efficacy and reducing systemic toxicity. Therefore, ApDCs take advantage of the small size of aptamers and deep tissue penetration ability to deliver payloads more effectively than conventional drug formulations.

Aptamers, hence, provide a highly customizable platform that bridges diagnostic recognition and therapeutic action in cancer immunotherapy. Through this article, we aim to critically examine the current landscape of aptamer-based immune modulators, including stand-alone aptamers and aptamer-drug conjugates, from the level of molecular engineering through preclinical development and toward clinical translation. We have also highlighted how selection strategies and structural design enable potent and specific anti-tumor immunity. Furthermore, we have discussed formulation, pharmacokinetic, and regulatory considerations that affect translational readiness. Finally, we explored the future perspectives of the aptamer technologies that can be applied toward a more prominent role in precision oncology as synthetic immune modulators that surpass the capabilities of the conventional immunotherapeutic methods presently available.

## 2 Fundamentals of aptamer technology

### 2.1 Structure, folding, and target recognition mechanisms

Aptamers are short, typically 20–100 nucleotide-long single-stranded nucleic acid sequences and have different functions based on their primary sequence and the intricate 3D shape adapted in a physiological environment. Usually, aptamers may exist as secondary structures with complementary base pairing, giving rise to common secondary motif structures like hairpin loops, G-quadruplexes, and bulges, among others. These secondary structures then further assemble to give rise to a compact and stable 3D confirmation that is stabilized in the presence of metal ions and interactions like base stacking. The final structure, hence, contains a specific binding pocket or a region that is highly specific to a target molecule. Aptamer technology makes the use of these short aptamer sequences to bind to particular targets like proteins with high affinity and selectivity (13), thus functioning like antibodies and providing enhanced stability and reduced immunogenicity. Aptamer binding is characterized by high selectivity and affinity that involves a combination of intermolecular forces and target recognition mechanisms (14). For instance, the predominant mechanism of interaction between the aptamer and target follows the induced fit model (15). According to this model, when the aptamer initially interacts with the target, both the components undergo minor conformational changes that optimize the fit and form a stable, high-affinity complex. This flexibility thus allows the aptamers to differentiate between closely related molecules. The final binding is mediated by several non-covalent interactions, including hydrogen bonding, Van der Waals forces, electrostatic, and hydrophobic interactions that ensure a strong and specific lock and key-like fit at the molecular level after induced fit (16). Owing to the small size and structural adaptability of aptamers, otherwise sterically inaccessible sites become accessible, thus allowing aptamers to modulate the biological function of the target. Hence, aptamer technology serves as a powerful tool for targeted drug delivery, diagnostics, and therapeutics, and plays a promising role in molecular medicine and research (17).

### 2.2 SELEX evolution and its recent innovations

**Figure 1:**
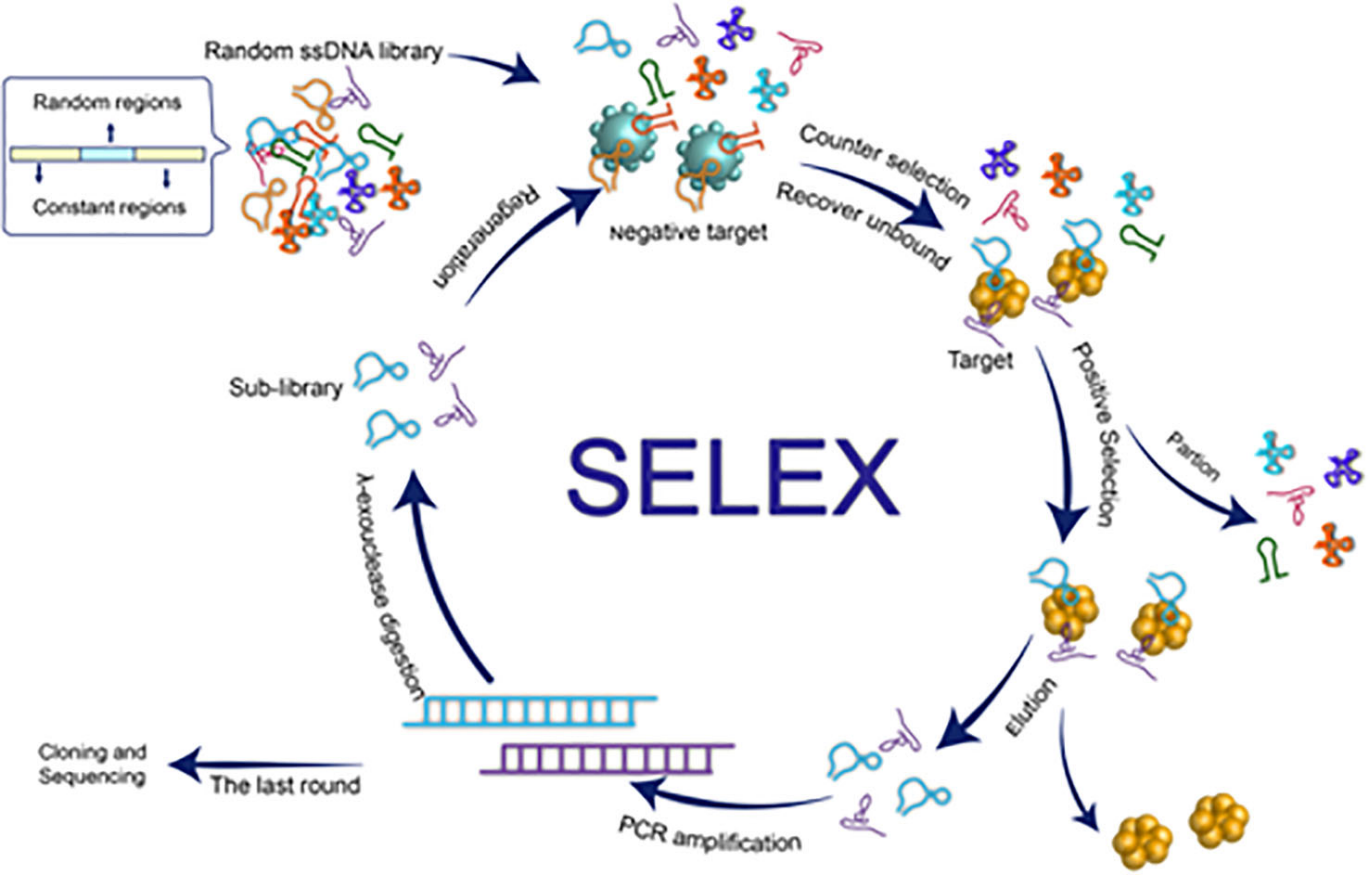
A schematic illustration of the aptamer generation by conventional SELEX process. Reproduced with permission from ref (27), copyright (2021) Frontiers.

Systematic evolution of ligands by exponential enrichment (SELEX) is used for screening high-affinity nucleic acid aptamers, as illustrated in **Figure 1**. SELEX is an iterative *in vitro* selection technique employed to isolate high-affinity and high-specificity nucleic acid aptamers from a large, randomized oligonucleotide library. The process begins with the incubation of the target molecule with a diverse pool of single-stranded DNA or RNA sequences, allowing specific binding interactions to occur. Non-binding sequences are subsequently removed through partitioning steps, while bound sequences are eluted and amplified using polymerase chain reaction (PCR) for DNA aptamers or reverse transcription-PCR for RNA aptamers. This cycle of binding, separation, and amplification is repeated multiple times to progressively enrich the pool with sequences exhibiting stronger affinity and specificity toward the target. As depicted in the figure, successive rounds of selection lead to the identification of optimal aptamer candidates with desired binding characteristics.

Different SELEX methods are used to adapt to various targets and improve the aptamer efficiency. Cell SELEX uses whole live cells as targets, thus allowing for aptamer selection that binds to targets in their native, functional conformations on the cell surface (18). Because this method does not need any previous knowledge of specific cell biomarkers or protein purification, it saves time and resources, thus helping discover new disease-specific biomarkers. Another method, the *in vivo* SELEX, also known as the tissue SELEX, selects aptamers within a living organism (19, 20). That is, a random aptamer library is injected into a model animal, and after a period of circulation, the target organ or tissue is harvested to extract the bound aptamers. This method is particularly advantageous as it allows for the aptamer selection that can cross biological barriers and function within the complex, dynamic environment of living tissues, and interact with the extracellular matrix and surrounding cells. Recently, AI-assisted SELEX, the *in-silico* SELEX, integrates computational tools and machine learning approaches to optimize the selection process and also predict aptamer-target interactions (21, 22). In this, bioinformatics tools are used to indicate the secondary and tertiary structures of the aptamers. Subsequently, molecular docking simulations were performed to estimate binding affinity, thereby guiding the experimental design. This approach can help significantly reduce the number of physical SELEX rounds needed, thereby saving time, cost, and labor. It also helps with handling the vast amounts of data generated by Next-Generation Sequencing (NGS), therefore enabling the identification of the best candidates earlier in the process (23). NGS allows for the monitoring of aptamer enrichment dynamics at every round, therefore enabling researchers to analyze millions of sequences and identify high-affinity binders much earlier than traditional Sanger sequencing (24). Other methods, like microfluidic and capillary electrophoresis SELEX, also help automate and miniaturize the SELEX process, thereby decreasing the required sample volumes, enhancing efficiency, and reducing the selection time from weeks to days (25, 26).

### 2.3 Chemical modifications for nuclease resistance and improved pharmacokinetics

To improve aptamer stability against nuclease degradation and to enhance their *in vivo* pharmacokinetics, chemical modifications in the sugar ring structure, phosphate backbone, and the terminal ends are carried out. Most commonly, sugar modification at the 2’ position is carried out by replacing the 2’-hydroxyl group with a 2’-fluoro, 2’-amino, or a 2’-O-methyl group to improve nuclease resistance and enhance the overall thermal stability of the resultant aptamer sequence (28). Alternatively, locked nucleic acids are used to build a methylene bridge between 2’-O and 4’-C of the sugar ring, thereby ‘locking’ the confirmation (29). Other methods, like terminal modifications or end capping, are performed to protect aptamer ends from exonuclease attacks (30). A common modification carried out is by using 3’-3’ inverted thymidine, in which the final nucleotide is linked in reverse orientation, thus effectively blocking the 3’ to 5’ exonuclease activity (31). Another similar method is connecting the 5’ to 3’ ends to form a circular shape, which thus disallows the exonuclease degradation and significantly improves the aptamer stability (32). The aptamer backbone structure is also modified by replacing a non-bridging oxygen atom in the phosphodiester linkage with a sulfur atom thus improving the nuclease resistance and hence enhancing the overall stability of the resultant aptamer.

### 2.4 Multivalency, linker chemistry, and conjugation principles

Often, multiple aptamer units are incorporated over a single construct to improve the overall binding strength, stability, selectivity, and functionality as compared to a monovalent aptamer sequence. Thus, multivalency helps improve the aptamer performance in biosensing and targeted therapies. The key principle of multivalency is cooperative binding, in which the binding of a single aptamer unit to the target promotes the binding of the adjacent units, thereby significantly reducing the overall dissociation rate. Aptamers can be integrated into multivalent constructs via covalent linkage, non-covalent assembly, or nucleic acid nanostructure self-assembly. Furthermore, for improved interaction between aptamers to a scaffold structure, linkers like varying length oligonucleotide structures (oligoT, oligoA, or oligoU), non-nucleotide linkers like polyethylene glycol, alkyl chains, or cleavable linkers are used. Thus, the design strategy for aptamer conjugation requires rigorous optimization as the optimal linker length and spatial arrangement are specific to each aptamer and target pair.

### 2.5 Aptamer-drug conjugates as an extension of aptamer engineering

Aptamer drug conjugates (ApDCs) are a promising field of aptamer engineering that merges aptamers with cytotoxic drugs for highly specific targeted therapy, especially in cancers (33). These conjugates can deliver potent payloads directly to diseased cells, thereby reducing harm to healthy tissues, unlike traditional chemotherapy (34). In addition, ApDCs offer advantages like rapid tissue penetration, high stability, and easier synthesis as compared to antibody drug conjugates (35). Therefore, this field builds on aptamer discovery by adding linkers and cytotoxic agents, thus allowing for programmable, multi-functional designs like multivalent or polymeric ApDCs for better drug loading and targeted release, hence marking a promising step toward personalized medicine.

A detailed comparison of aptamers, antibodies, and peptides in cancer immunotherapy have been discussed in **Table 1**. As can be inferred from the table, although aptamers are generally considered to have low immunogenicity compared to protein-based biologics, emerging evidence suggests that certain chemical modifications, including 2’-modified nucleotides, locked nucleic acids, and PEGylation, may introduce immunogenic responses in specific contexts (36, 37). Moreover, increasing structural complexity through multivalent designs or linker chemistries may elevate manufacturing costs, narrowing the economic advantage traditionally associated with aptamers. Therefore, while aptamers retain favorable immunological and production profiles, these factors should also be considered when evaluating next-generation constructs.

**Table 1:** Comparison of aptamers, antibodies, and peptides in cancer immunotherapy.

Parameter	Aptamers	Antibodies	Peptides	Ref.
Molecular size	Small oligonucleotides (~10 to 30 kDa)	Large proteins (~150 kDa, IgG)	Small amino-acid chains (~1 to 5 kDa)	(175, 176)
Structural complexity	Single-stranded DNA/RNA forming defined 2°/3° structure	Heterotetrameric glycoproteins with 2 heavy and 2 light chains	Linear or cyclic peptides, simpler than proteins	(177)
Binding affinity	High (often low nM to pM)	Very high (typically pM to nM)	Generally lower (nM to µM)	(176, 178)
Target specificity	Very high, SELEX‐selected for unique targets	Very high, antigen-specific	Moderate to high, designed against cell-surface receptors	(5, 15)
Stability (*in vitro*/*in vivo*)	*In vitro*: Good if properly folded; *In vivo*: Poor when unmodified	Generally stable in serum; require cold storage (–20 °C) to avoid denaturation	*In vitro*: Moderate; *In vivo*: Poor, results in rapid protease degradation	(176, 179, 180)
Immunogenicity	Very low as are non-protein so minimal immune response	Can be immunogenic	Low as small sized and are often self-derived	(181)
Tumor penetration	Good as small size enhances tissue and tumor penetration	Poor as large size leads to perivascular localization	Good as small size results in deep and rapid penetration	(176, 182)
Circulatory half-life	Short (minutes), unmodified are rapidly renal cleared, can be extended to hours with modification, Eg: PEGylation	Long (days to weeks), Eg: IgG ~3 weeks	Short (hours), rapid clearance	(5, 176)
Ease of chemical modification	High as are synthetic and can be easily labeled or chemically modified	Low as requires protein engineering or fusion	High as solid-phase synthesis allows diverse modifications	(176)
Manufacturing scalability and cost	Moderate: fully chemical synthesis, easy quality control, lower costs	Challenging: cell-culture production, high purification cost	High: chemical synthesis, low cost, easily scaled	(183, 184)
Advantages	Small, low-immunogenic, reversible folding, high specificity, easy and scalable synthesis	Extremely high affinity and specificity, long half-life	Small, well-tolerated, deep tissue penetration, tunable specificity, low cost and toxicity	(176, 185)
Limitations	Rapid renal clearance, vulnerable to nuclease degradation	Large size, poor penetration, potential immunogenicity, high cost, systemic toxicities	Rapid proteolysis, short half-life, typically lower affinity	(5, 30, 185, 186)

## 3 Mechanistic classes of aptamer-based immunotherapies

The main mechanistic classes of aptamer-based immunotherapies are based on the high specificity of aptamers to either directly modulate immune responses or to deliver therapeutic payloads to target cells (5). This is achieved by immune checkpoint inhibition, cytokine modulation, cellular immunotherapy, bispecific aptamers, aptamer-drug conjugates, and nanoplatforms (38).

### 3.1 Checkpoint blockade and immune activation

**Figure 2:**
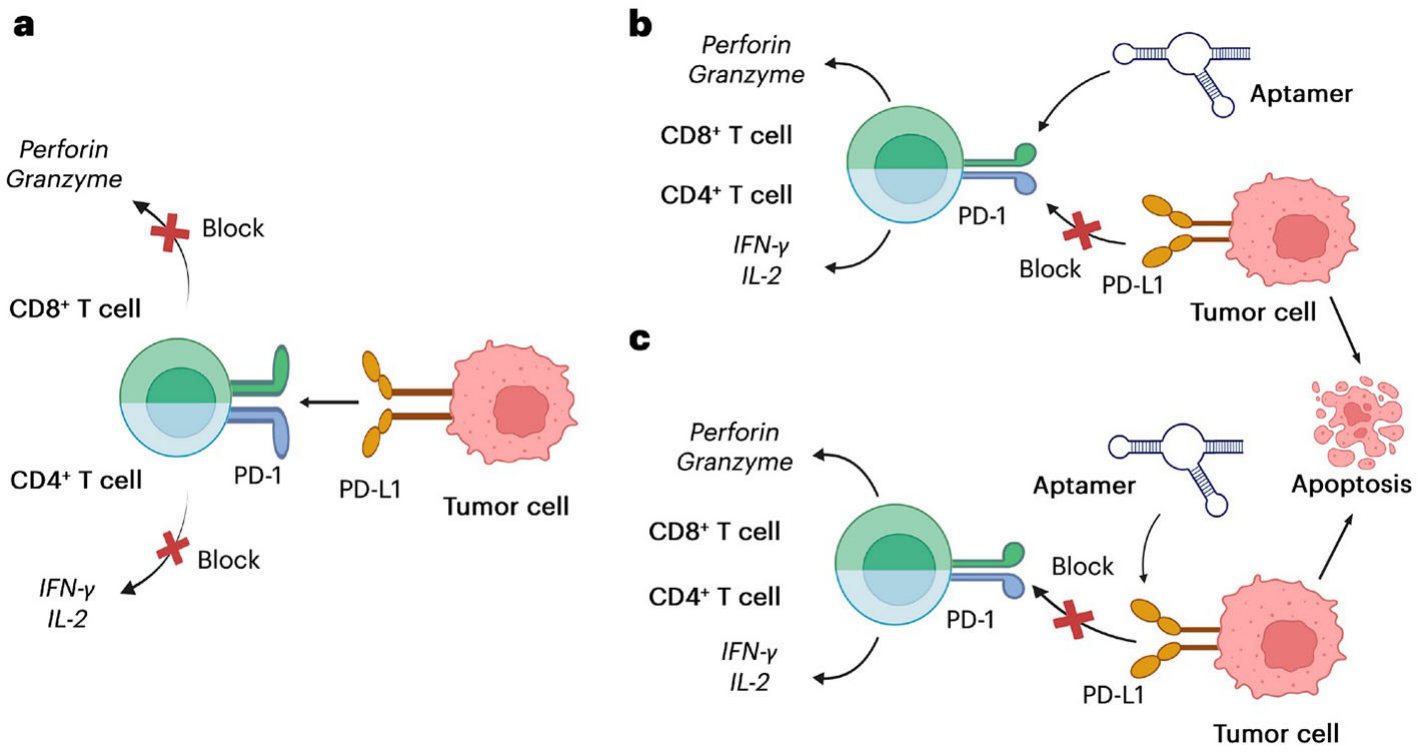
The PD-1/PD-L1 route of action and the use of an aptamer to target PD-1/PD-L1 to block their interaction. **(a)** PD-1 binds to PD-L1 and an inhibitory signal is transmitted to the T-cell, suppressing the immune response and allowing the tumor cell to evade immune detection. **(b, c)** Aptamers bind to either PD-1 on T-cell surface or PD-L1 on tumor surface, blocking the PD-1/PD-L1 interaction, restoring T-cell immune activity, and releasing cytotoxic agents to promote cancer cell destruction. Reproduced with permission from ref (1), copyright (2025) MDPI.

Aptamer ligands can be engineered to block immune checkpoints on T cells and tumor cells, thereby establishing anti-tumor activity. For instance, aptamers targeting PD-1 or PD-L1 have been shown to bind to the targets with high affinity and block the PD-1/PD-L1 interactions, thus causing an enhanced T-cell proliferation and cytokine production, as shown in **Figure 2** (1). Under physiological and tumor-associated conditions, the interaction between PD-1 expressed on T cells and PD-L1 expressed on tumor cells delivers an inhibitory signal that suppresses T-cell activation, thereby facilitating immune evasion by cancer cells (**Figure 2a**). Aptamers designed to specifically target either PD-1 or PD-L1 have demonstrated high binding affinity and functional efficacy in disrupting this immune checkpoint interaction. As illustrated in **Figures 2b, c**, these aptamers competitively bind to PD-1 on the T-cell surface or PD-L1 on tumor cells, effectively blocking their interaction. This blockade alleviates the inhibitory signaling cascade, leading to the restoration of T-cell proliferation, activation, and cytokine production. Consequently, the reactivated immune cells regain their cytotoxic potential, promoting targeted tumor cell destruction.

Another CTLA-4 binding aptamer has been developed that prevents CTLA-4 from engaging its B7 ligands, hence reducing T cell activity and improving the effector T cell responses (39). Recent studies have also focused on other checkpoints, like the LAG-3 targeted aptamers that release LAG-3-mediated inhibition of T cells (5, 40, 41). In certain preclinical models, such checkpoint aptamers have shown synergistic effects when used in combinations. For instance, bispecific aptamers that target both PD-1 and CTLA-4 can simultaneously block multiple pathways and also recruit immune effectors (1). Moreover, the small size and modularity of aptamers also allow for localized immune activation. Upon coupling aptamers with tumor-targeting elements or cytokines, immune stimulation can be confined to the tumor microenvironment (5).

### 3.2 Immune-cell redirection and co-stimulation

Apart from playing a crucial role in immune response, aptamers can also actively direct and stimulate immune cells. Certain co-stimulatory receptors on T cells, like CD28, 4-1BB, and OX40, can be targeted by agonistic aptamers. For instance, in a recent study, a DNA aptamer specific for CD28 was isolated and was shown to bind CD28 on T cells. As a monomer, it could block the natural CD28-B7 co-stimulatory interaction; however, when the same aptamer was dimerized, it could function as an artificial co-stimulatory ligand (42). Similarly, an RNA aptamer against 4-1BB has also been developed (43). When presented in a multivalent form, it can co-stimulate CD8+ T cells *in vitro* and promote tumor rejection. Other studies have also reported agonistic OX40 aptamers that result in intratumoral CD8+ T cell proliferation, cytokine release, and suppressed regulatory T cells, which in turn induced tumor regression even at distant sites (44). Aptamers have also shown to redirect immune effector cells to tumors, thus mimicking bispecific T-cell engagers that can result in immune synapses and potent cytotoxicity (5, 45).

### 3.3 Cytokine and immune-suppressive pathway modulation

Aptamers can also be used to modulate soluble factors that regulate the immune response by targeting immunosuppressive cytokines or enzymes within the tumor cells. Such aptamer-directed siRNA can neutralize TGF-β signaling in T cells (46). DNA aptamer can also bind and inhibit indoleamine 2, 3-dioxygenase 1 enzyme that depletes tryptophan and generates immunosuppressive metabolites (47). Aptamers have been developed for other cytokine pathways, like the anti-angiogenic aptamer pegaptanib that binds the VEGF-165 isoform, blocking its signaling (48). Such VEGF-targeting aptamers can normalize tumor vessels and help overcome VEGF-mediated immune suppression, hence improving T-cell infiltration. Moreover, aptamers can also deliver pro-immune cytokines or inhibitors of suppressive signals in a site-specific manner, thereby helping fine-tune the balance of stimulatory versus inhibitory effects in the tumor microenvironment (5).

### 3.4 Tumor microenvironment reprogramming

Recently, aptamers have also been applied as nucleic acid carriers to reprogram tumor-associated cells and thus aid in normalizing tissue microenvironment (46). Aptamers can also be linked to anti-miRs or miRNA sponges to alter tumor gene expression. This was seen in a recent study where a chimeric molecule was developed by combining an Axl‐targeting aptamer with a miR-214 sponge (49). The Axl aptamer could guide the conjugate to Axl+ breast cancer cells, thus helping to aid in a significant reduction of cancer cell migration and invasion. Therefore, aptamer-anti-miRNA conjugates can be applied to neutralize oncogenic miRNAs in a targeted way.

Aptamer strategies can also be applied to help overcome other tumor-associated conditions like tumor hypoxia, which is a key driver of immunosuppression and therapy resistance. This can be achieved by using a triple negative breast cancer (TNBC)-targeting aptamer that can deliver a self-assembled nanomedicine. Upon tumor localization, the aptamer-carrier releases a hypoxia-activating prodrug along with an antisense oligonucleotide against HIF-1α (50). In hypoxic tumor regions, the prodrug is converted to a cytotoxin, while the anti-HIF-1α ASO hinders HIF-1α–mediated drug resistance (50). Thus, aptamers can mediate both targeting and multi-modal reprogramming of the hypoxic microenvironment. Similarly, aptamers can also be selected or modified such that they penetrate dense stroma.

Thus, in combination, these tumor and environment-specific aptamer constructs can recondition the tumor microenvironment, thereby turning them from a suppressive niche into a more immune-permissive environment (50, 51).

### 3.5 Aptamer-drug conjugates as immunomodulatory therapeutics

**Figure 3:**
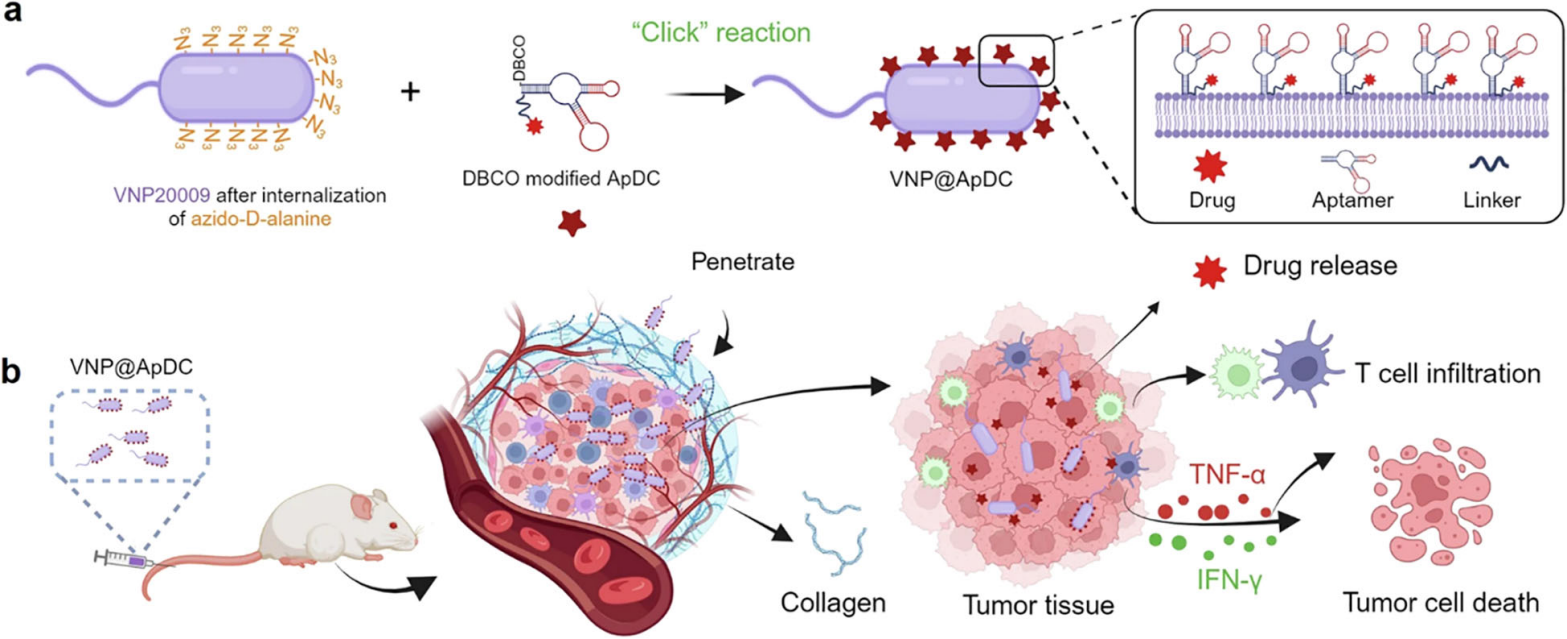
Schematic illustrating the construction of functionalized bacteria and the mechanism against pancreatic tumors. **(a)** Preparation of ApDC-anchored drug via a simple one-step click chemistry process. **(b)** Drug-loaded bacteria penetrate the stromal barriers of pancreatic neoplasms after intravenous administration, effectuating deep-tissue drug release. Concurrently, they secrete cytokines, triggering both apoptosis and necrosis in malignant cells, while amplifying the recruitment of immune cells to the tumor site. Reproduced with permission from ref (52), copyright (2024) Nature.

As discussed in previous sections, aptamer-drug conjugates (ApDCs) integrate targeted chemotherapy with immune modulation. In an ApDC, the aptamer guides a cytotoxic drug to the tumor, concentrating the kill signal, while the payload activates immunity (52), as shown in **Figure 3**. As can be seen in **Figure 3a** the construction of the ApDC has been depicted through a streamlined one-step click chemistry approach, wherein the aptamer is conjugated to the therapeutic payload to form a stable and functionalized complex. This strategy preserves the targeting capability of the aptamer while ensuring efficient drug loading. Following systemic administration, as shown in **Figure 3b**, the ApDC-loaded bacteria exploit their inherent tumor-homing properties to penetrate the dense stromal barriers characteristic of pancreatic neoplasms. This enables deep tumor infiltration and localized drug release, thereby enhancing the concentration of the cytotoxic payload at the tumor site while minimizing off-target effects. In parallel, the bacteria act as bioactive carriers that secrete immunostimulatory cytokines, promoting both apoptotic and necrotic cell death in malignant tissues. This dual action amplifies direct tumor cell killing and enhances immune cell recruitment and activation within the tumor microenvironment. For instance, the AS1411 aptamer that binds nucleolin on cancer cells has been used to deliver doxorubicin specifically to tumor cells (53). In a recent study, a molecular hybrid of AS1411, an anti‐hTERT antisense, was created with doxorubicin. The resultant multi-functional conjugate displayed potent anti-proliferative effects, that is, doxorubicin loading decreased expression of hTERT and vimentin, downregulated Bcl-2, and upregulated Bax, and induced cancer cell apoptosis (54). Similar activity was observed in another study where an EpCAM-specific aptamer was conjugated to doxorubicin to target colorectal cancer stem cells. The EpCAM‐Apt-DOX was observed to significantly increase the tumor growth inhibition and reduce cancer stem cell frequency in mice (55). Therefore, through the ApDCs, the aptamer improves drug delivery, increases tumor cell uptake, and cytotoxicity relative to the untargeted drug. Apart from direct cytotoxicity, ApDCs can also induce immunogenic cell death, thereby indirectly facilitating the anti-tumor immunity (56). Thus, ApDCs can also be engineered in combination forms by integrating a cytotoxin with a checkpoint blockade aptamer, a toll-like receptor (TLR) ligand, or other immune modulators to achieve both direct tumor kill and immune priming.

## 4 Aptamer-based delivery platforms in immunotherapy

### 4.1 Nanoparticle-linked aptamer systems

Nucleic acid aptamers are commonly conjugated to various nanocarriers, like gold nanoparticles (AuNPs) (57), liposomes (58), magnetic nanoparticles (59), quantum dots (QDs) (60), and DNA origami nanostructures (61) to achieve targeted delivery of therapeutics to tumors or immune cells. For instance, AuNPs-aptamer conjugates can be applied both for targeted imaging and radiosensitization of tumor cells (62, 63). In addition, aptamer-decorated liposomes can carry chemotherapeutics or siRNAs directly to tumor cells. Aptamers like TIM-3 on liposomes can be applied to deliver checkpoint inhibitors and drugs simultaneously (1). Likewise, superparamagnetic NPs functionalized with LAG-3 aptamers allow targeted photothermal or magnetic hyperthermia treatment (64, 65). Other systems, like DNA-origami nanostructures folded into 3D shapes, can display multiple aptamers for multivalent targeting or encapsulate payloads for controlled release (66). Such aptamer-integrated origami systems have shown improved stability and precise, multi-functional delivery (67). Other polymeric NPs like poly(lactic-co-glycolic acid) (68, 69), chitosan (70, 71), and silica NPs (72) have also been functionalized with aptamers for tumor targeting. These aptamer-nanocarrier conjugates integrate the high drug-loading capacity and enhanced tissue penetration of nanoparticles with the molecular specificity of aptamers and thus are promising for therapeutic applications.

### 4.2 Aptamer-drug conjugates

Aptamers can be chemically linked to cytotoxic or immunomodulatory payloads to form ApDCs, like antibody-drug conjugates (ADCs), as discussed in detail in **Table 2**. Compared to ADCs, ApDCs offer several distinctive advantages. Firstly, because of the small size of the aptamers, the resultant ApDCs can easily penetrate deeper layers of the tumor tissues (73). Moreover, as aptamers are synthetic oligonucleotides, they allow for a facile chemical synthesis and permit site-specific modifications to attach multiple drug moieties (74). Aptamers also tend to be more chemically stable and less immunogenic than antibodies (75). In addition, the ~2-nm hydrodynamic size of an aptamer allows more uniform distribution in solid tumors as compared to ADCs (76). The chemical nature of aptamers allows for ApDC manufacture via solid-phase synthesis in high purity, avoiding protein manufacturing (30, 77). Owing to the promising features of aptamers, dual-payload ApDCs can be developed, that is, aptamers can be loaded with two distinct payloads that include a cytotoxic drug and an immune adjuvant. Such a dual action ApDCs would allow for killing of tumor cells along with simultaneously priming anti-tumor immunity (78). In addition, as with ADCs, ApDCs also use cleavable linkers to release their payloads under tumor-specific conditions. Usually, pH-sensitive, enzyme-sensitive, or redox-sensitive linkers are all applied in ApDC design to ensure controlled release of the drug inside the target cells. Such stimuli-responsive linkers thus help reduce systemic toxicity and help concentrate the active drug at the disease site. More importantly, ApDCs demonstrate an inherent capacity to synergize with immunotherapy by inducing immunogenic cell death and antigen spreading (78). Therefore, by combining targeted killing with local immune stimulation, ApDCs can amplify antigen presentation, T-cell priming, and anti-tumor immunity, potentially enabling antigen spreading beyond the original target.

**Table 2:** Key aptamer-drug conjugates (ApDCs) in cancer immunotherapy.

ApDC	Target antigen	Aptamer type	Drug payload	Formulation	Key outcomes	Ref.
sTN145 aptamer/PD-L1 siRNA	Triple-negative breast cancer (TNBC) cells	RNA	PD-L1 siRNA	PLGA-PEG nanoparticle loaded with siRNA	Efficient and selective PD-L1 knockdown in TNBC cell; no uptake in non-TNBC cells	(187)
PA9–1 aptamer/Antisense oligonucleotide (ASO)	PD-L1	DNA	Antisense oligonucleotide	Free aptamer-ASO chimera	Dual-function: aptamer blocks PD-1/PD-L1 interaction and delivers ASO to silence PD-L1 expression	(78)
Sgc8c aptamer/Monomethyl auristatin E	Protein Tyrosine Kinase 7	DNA	Monomethyl auristatin E (MMAE)	Free ApDC	Sustained tumor regression in multiple PTK7+ xenografts, high tumor MMAE accumulation with rapid normal-tissue clearance	(107)
AptB1/Cisplatin	EAAT2/Nucleolin (NCL)/YB-1	DNA	Cisplatin	Free ApDC	AptBCis1 gave superior tumor suppression; achieved targeted delivery and potent anti-tumor effects	(188)
HER2 aptamer/DM1	HER2	RNA	DM1	Free ApDC	HER2-targeted ApDC bound selectively to HER2+ cells, significantly inhibited BT-474 tumor growth with minimal weight loss or toxicity	(189)

### 4.3 Aptamer-CAR-T hybrid systems

Aptamers have also been integrated into chimeric antigen receptor (CAR) designs to create flexible, non-genetically modified CAR-T cells (79). In a recent study, Zhou et al. CAR aptamers were constructed by replacing the scFv of a conventional CAR with an aptamer that recognizes a tumor antigen (80). These Apt-CAR T cells could be directed to kill target cells in the presence of a free aptamer or an aptamer-antigen linkage, thus allowing T-cell specificity to be switched by the aptamer trigger. The resultant aptamer-based CAR showed tunable antigen-binding avidity and could be engineered to respond to the tumor microenvironment. Therefore, this non-genetic approach avoids permanent gene editing of T cells; that is, the T-cell activity can be effectively programmed by administering soluble aptamers or aptamer-coated tumor binders. Thus, the study confirmed that the developed Apt-CAR T cells can be directly activated by aptamer-bound cancer cells and can kill them both *in vitro* and *in vivo*.

A key distinction between aptamer-based CAR systems and conventional genetically engineered CAR-T cells lies in their durability and persistence. Unlike traditional CAR-T therapies, which rely on stable genetic modification and long-term *in vivo* expansion, aptamer-mediated CAR systems are inherently transient and require the continued presence of the aptamer for sustained activity (81). While this may limit long-term persistence in high-turnover tumor environments, it offers a significant advantage in terms of controllability and safety, allowing real-time modulation or termination of activity by adjusting aptamer dosing. Thus, Apt-CAR systems may be better positioned as a tunable and potentially safer alternative, particularly in settings where precise temporal control of immune activation is desirable.

### 4.4 Aptamer-guided delivery of mRNA, siRNA, or CRISPR to immune or tumor cells

Aptamers also guide the delivery of genetic therapies; this can be achieved by applying lipid or nanoparticle formulations decorated with immune cell-targeting aptamers to deliver mRNA or siRNA selectively (81). For instance, a recent study demonstrated the use of a T-cell–targeting aptamer to ferry OX40 mRNA into murine splenocytes. The conjugation of OX40 mRNA to a CD4/CD8-binding aptamer increased its uptake about 4 to 7-fold in CD4+ and CD8+ T cells, boosting transfection efficiency significantly over untargeted mRNA (82). Similarly, in another study, an AS1411-aptamer-functionalized lipid NP was used to deliver PD-L1 siRNA into A549 lung carcinoma cells, resulting in PD-L1 knockdown, enhanced T-cell activation, and tumor-cell apoptosis (83).

Aptamers have also been used to deliver CRISPR/Cas9 cargoes via aptamer-conjugated polymeric or viral-like particles that can carry Cas9-sgRNA complexes to tumor cells for gene knockout (74, 81, 84). In principle, aptamer-guided CRISPR can allow for targeted gene editing of oncogenes or immune-checkpoint genes in the tumor microenvironment. Thus, by choosing appropriate aptamers and payloads, one can deliver small molecules, antisense RNAs, siRNAs, or specifically to tumors or immune subsets (82). Therefore, this versatility makes aptamers promising vehicles for next-generation nucleic-acid immunotherapies.

### 4.5 Pharmacokinetics and biodistribution optimization of aptamer constructs

Unmodified aptamers are rapidly cleared from circulation due to their small size, nuclease degradation, and renal filtration (85). To overcome this, aptamers can be chemically modified to resist nucleases. In addition, aptamers can be functionalized with bulky conjugates like polyethylene glycol (86), cholesterol (87), and albumin-binding peptides (88) to avoid rapid clearance and to prolong circulation. Furthermore, conjugation of aptamers to liposomes (58) or nanoparticles (89, 90) increases molecular weight and allows for enhanced permeability and retention driven tumor accumulation and thus helps optimize the resultant aptamer systems.

## 5 Preclinical and clinical translation

**Figure 4:**
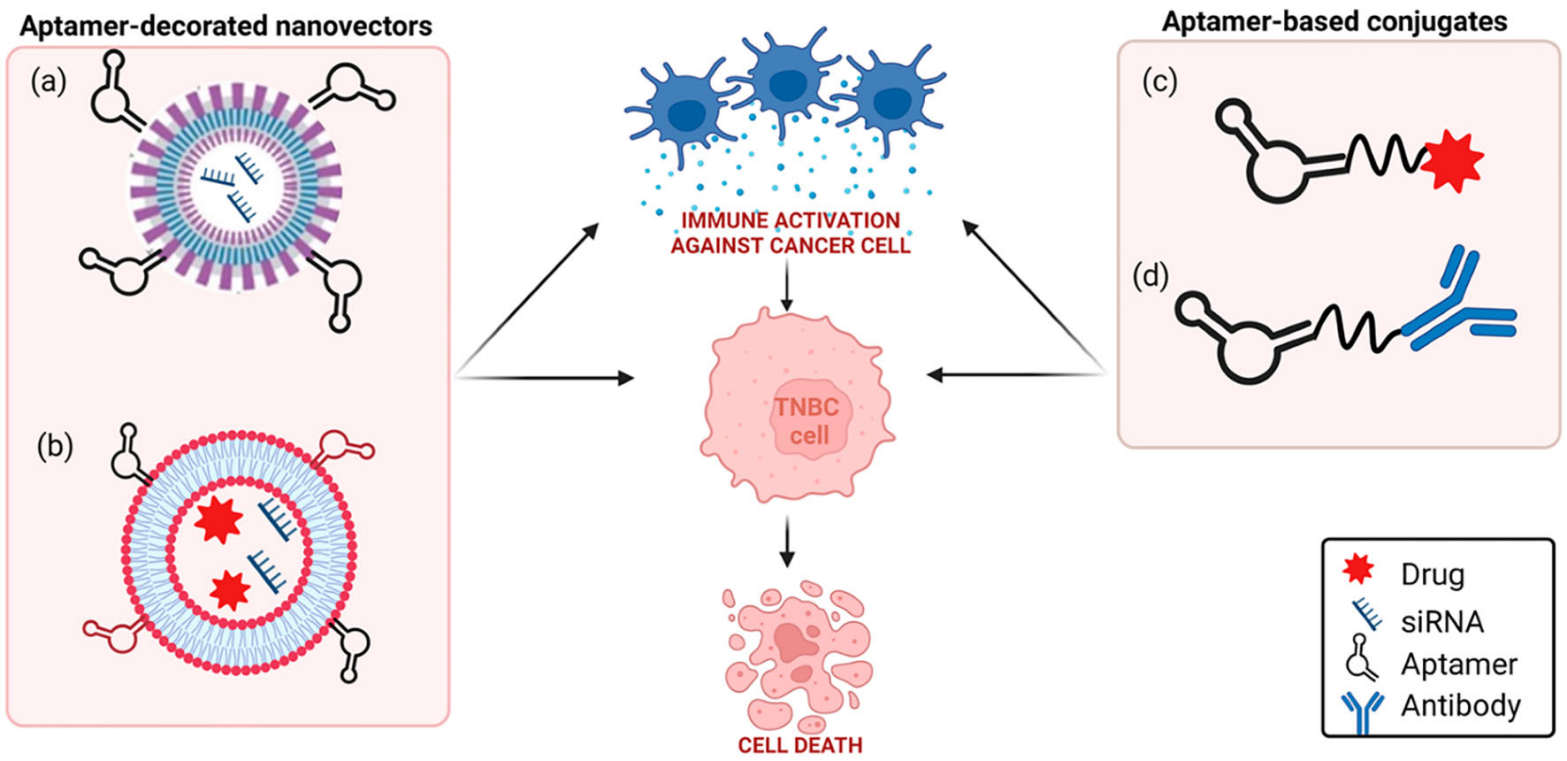
Schematic representation of aptamer-based strategies to block PD-1/PD-L1 axis in TNBC. **(a)** TNBC aptamer-decorated nanoparticles loaded with anti-PD-L1 siRNA; **(b)** anti-CD44 and anti-PD-L1 aptamer-decorated liposomes loaded with both doxorubicin and anti-IDO1 siRNA; **(c)** anti-PD-L1 aptamer conjugated to paclitaxel; **(d)** anti-EGFR aptamer covalently linked to anti-PD-L1 or anti-CTLA-4 mAbs. Reproduced with permission from ref (11), copyright (2023) MDPI.

Multiple preclinical studies have been carried out in recent years that successfully demonstrate the potency of aptamers as immunotherapeutics. For instance, in a recent study by Jiang and colleagues in murine cancer models, PD-1-specific DNA aptamers were shown to effectively block the PD-1/PD-L1 interaction, leading to increased cytokine release and robust CD8+ T-cell proliferation (11, 91), as also shown in **Figure 4**. As illustrated in **Figure 4a**, TNBC-targeting aptamer-decorated nanoparticles are employed for the delivery of anti-PD-L1 siRNA, enabling gene silencing of PD-L1 expression and subsequent immune checkpoint blockade. **Figure 4b** further expands this concept by incorporating dual-targeting aptamers (anti-CD44 and anti-PD-L1) on liposomal carriers loaded with both doxorubicin and anti-IDO1 siRNA, thereby integrating chemotherapy with immunomodulation and metabolic pathway inhibition. In **Figure 4c**, a direct conjugation approach is depicted, where an anti-PD-L1 aptamer is linked to paclitaxel, facilitating targeted cytotoxic delivery while simultaneously blocking immune suppression. Lastly, **Figure 4d** demonstrates a hybrid strategy in which an anti-EGFR aptamer is covalently conjugated to immune checkpoint monoclonal antibodies, combining the targeting precision of aptamers with the established efficacy of antibody-based immunotherapy.

Furthermore, DNA aptamers directed against CTLA-4 have also been shown to inhibit regulatory T-cell function and slow melanoma growth in mice (39). Importantly, these aptamers restore T-cell activity without the high immunogenicity of protein antibodies (1, 92). Beyond regulating the checkpoints, bispecific aptamers and immune agonists can also be developed to activate anti-tumor immunity directly. Bispecific constructs that physically link a tumor antigen to an immune-cell receptor can form artificial immune synapses that trigger cytotoxic killing (93). Moreover, aptamers against co-stimulatory receptors like CD28, OX40, and 4-1BB can also be used to improve T-cell activation in combination with checkpoint blockade (93).

Certain aptamer therapeutics, as explored in recent studies, have also advanced into clinical trials, confirming the translational potential of the platform, as critically discussed in **Table 3**. A nucleolin-binding DNA aptamer, AS1411, was among the first anti-cancer aptamers tested (94, 95). The AS1411 reached phase II trials for acute myeloid leukemia and metastatic renal cell carcinoma, exploiting the overexpression of nucleolin on cancer cell surfaces (96). Pegaptanib (Macugen) is a PEGylated RNA aptamer that binds VEGF-165 and was FDA-approved in 2004 for wet age-related macular degeneration (1, 97, 98). Another such promising candidate is NOX-A12, an L-RNA aptamer that antagonizes the chemokine CXCL12/SDF-1 and has progressed into mid-stage clinical trials for glioblastoma and other solid tumors (99). By blocking CXCL12, NOX-A12 can disrupt tumor-stromal interactions that promote resistance and metastasis (100).

**Table 3:** Summary of the different recent clinical stage aptamers.

Agent and trial	Developer	Aptamer type	Target	Phase	Safety effects	Formulation	Ref.
AST-201	Aptamer Sciences Inc. (South Korea)	DNA aptamer targeting GPC3, with integrated gemcitabine residues	Glypican-3 on tumor cells	I	Moderate toxicity	Intravenous infusion of gemcitabine-linked aptamer	(190)
AM003	Aummune Ltd. (Israel)	Multimodal individualized DNA aptamer	Bispecific as one arm engages tumor antigen, other engages T-cells	I	Generally mild, moderate	Local intratumoral injection of aptamer	(191, 192)
Olaptesed Pegol (NOX-A12)-CLL	TME Pharma (NOXXON Pharma AG, Switzerland)	PEGylated L-RNA Spiegelmer binding CXCL12	Chemokine CXCL12	IIa	No DLTs	IV infusion of Spiegelmer before each chemo cycle	(193, 194)
Olaptesed Pegol (NOX-A12)-Multiple Myeloma	TME Pharma (NOXXON)	PEGylated L-RNA Spiegelmer	CXCL12	IIa	Safe and well tolerated	IV aptamer before chemotherapy	(195)

### 5.1 Translational barriers

Despite the promising potential of aptamer therapeutics, there are several key obstacles that need to be overcome for their clinical application. For starters, chemical and metabolic stability are primary concerns. Unmodified DNA or RNA aptamers are rapidly degraded by nucleases in blood, thus having a very low half-life of minutes. To overcome this, therapeutic aptamers can be chemically modified, for instance by integrating 2’-fluoro or 2’-O-methyl substitutions on the ribose sugar, phosphorothioate backbones, or unnatural nucleic acids to resist nuclease attack (101). More commonly, capping of the 3’ end is carried out, or modified bases are incorporated to further block the exonuclease activity (30). Such modifications thus help improve aptamer stability by 10 times or more and help to preserve binding affinity and avoid toxicity. Another essential factor that needs to be considered is the off-target binding. Even with good target affinity, some aptamers can exhibit non-specific interactions by binding to serum proteins or adhering to cell surfaces in a charge-dependent manner (102, 103). These off-target bindings can significantly reduce effective drug concentration and potentially cause side effects like platelet aggregation or complement activation (76, 92). Thus, rigorous SELEX and counter-selection steps are important to minimize cross-reactivity.

To minimize the off-target interactions, rigorous selection and validation pipelines are required beyond conventional SELEX. Counter-SELEX against abundant serum proteins such as albumin, immunoglobulins, and complement factors should be incorporated early in the selection process to eliminate sequences with nonspecific binding (104). In addition, cell-based negative selection against non-target primary cells and stromal components can further improve the specificity (105). Post-selection validation should also include plasma protein binding assays, competitive binding studies, and *in vivo* biodistribution analyses using labeled aptamers (106). Thus, these approaches provide a systematic framework to reduce cross-reactivity and improve the therapeutic index of aptamer-based constructs.

Furthermore, new aptamer candidates must be screened against panels of non-target cells and tissues to confirm specificity, and any unintended binding or tissue cross-reactivity must be characterized preclinically. More importantly, the small size of aptamers often leads to very rapid kidney filtration and urinary excretion, resulting in a short systemic half-life, usually only a few hours or less for unmodified aptamers (101). To prolong aptamer circulation, a bulky moiety like polyethylene glycol can be added to extend half-life to several hours. Additionally, multivalent aptamers can be developed by conjugating aptamers to albumin-binding domains or by functionalizing them with nanoparticles. These methods can thus help increase the effective molecular weight beyond the renal filtration threshold (107).

More importantly, the fundamental design trade-off in aptamer therapeutics lies between pharmacokinetic optimization and tissue penetration. While the small molecular size of aptamers allows for a superior tumor penetration compared to antibodies, it also leads to rapid renal clearance and short circulation half-life (108). Chemical modifications such as PEGylation, cholesterol conjugation, or albumin-binding domains increase the hydrodynamic radius and prolong systemic exposure but may partially compromise deep tumor penetration. However, recent studies also suggest the existence of an optimal size window in which renal filtration is reduced while preserving sufficient tissue diffusion (109, 110). To address this balance, alternative strategies such as reversible PEGylation (111), albumin hitchhiking (88), and nanoparticle-assisted delivery (112) are being explored to decouple circulation time from tissue penetration. These approaches aim to preserve one of the core advantages of aptamers while enabling clinically relevant pharmacokinetics.

### 5.2 Translational insights from antibody-drug conjugate development for guiding aptamer-drug conjugates translation

The maturation of antibody-drug conjugate (ADC) therapies over the past decade offers valuable translational considerations for developing aptamer-drug conjugates (ApDCs). For starters, only ultra-potent cytotoxins have been noted to yield sufficient efficacy at safe dosing using ADCs (113–115). Similarly, ApDCs also require strong payloads (107). Moreover, the linker used in ApDCs must be stable in circulation but cleavable in the tumor environment, which can be achieved using proteases or pH change. Integrating a proper linker design prevents premature drug release, thus ensuring there is no off-tumor toxicity while ensuring efficient release inside the cancer cells. In addition, for maximum therapeutic effects, ApDCs must be developed with high target expression and cancer-specific antigen selectivity, similar to ADCs (107). Thus, aptamers against a well-validated tumor marker must be selectively chosen such that. To achieve this, bispecific aptamers that have dual checkpoint targeting features or multivalent aptamers can be considered for high selectivity. Furthermore, dose schedules and payload limits must also be considered to avoid toxicity resulting from ApDCs. Studies have shown that ApDCs can achieve ADC-like potency with a greater therapeutic window, given their rapid clearance (107). Therefore, careful dose escalation and toxicity monitoring of ApDCs are crucial to achieve their full potential in cancer therapy.

## 6 Computational and systems design

### 6.1 Machine learning and AI in SELEX optimization and drug conjugate prediction

Recent advances in computational biology have revolutionized the design of aptamers and ApDCs for cancer immunotherapy. Machine learning (ML) and artificial intelligence (AI) can now be integrated with wet-lab SELEX techniques, thereby dramatically accelerating aptamer discovery and optimization. ML-guided SELEX approaches can use affinity-sorted libraries to train predictive models. This was observed in a study by Bashir et al., where the team trained a neural network on particle-display SELEX data that enabled *in silico* screening of aptamer variants with ~11-fold enrichment of high-affinity binders (116). Similarly, in another study by Yang et al., a DeepAptamer hybrid CNN/LSTM model was trained on early SELEX reads that could rapidly identify binding motifs and prioritize high-affinity candidates *in silico* with high efficiency (117). The DeepAptamer could efficiently predict sequences that showed nanomolar binding without additional repetitive rounds.

Furthermore, beyond predictive ranking, generative AI can also propose entirely new aptamer sequences. Wang and colleagues introduced AptaDiff, a diffusion-based generative model that can create aptamers from learned sequence-affinity landscapes (118). The Bayesian optimization by AptaDiff could produce novel aptamers with 2 to 3 times lower KD values than the best SELEX candidates, as further validated by surface plasmon resonance. Another recent study by Gupta et al. described an AIoptamer in which known aptamer-target complexes can be mutated computationally and screened through AI affinity predictors, PredPRBA and PDA-Pred, before structural modeling and molecular dynamics optimization (119).

However, despite the promising potential of AI and ML in SELEX optimization, there are a few limitations that still need to be considered. For starters, specific models require extensive high-quality training data and may not generalize across all target classes (120). Moreover, ML models typically predict affinity scores without any mechanistic insight. In addition, a critical limitation remains in their reliance on affinity prediction without mechanistic interpretability (117, 121). To overcome this shortcoming, a hybrid pipeline integrating computational prediction with structural and biophysical validation is needed. Therefore, in such a framework, AI derived sequences can be first subjected to secondary and tertiary structure prediction followed by molecular docking and molecular dynamics stimulations to assess the binding stability, conformational flexibility, and potential off target interactions (122, 123). These in silico predictions must then be experimentally validated via orthogonal techniques like surface plasmon resonance or isothermal titration calorimetry to quantify binding kinetics along with mutational scanning to assess the functional motifs (124). In addition, integrating explainable AI approached with structural modeling can help identify sequence structure function relationships instead of solely relying on black box affinity scores (125, 126).

Another major limitation in current AI-assisted SELEX workflows is the lack of standardized, high-quality training datasets. To improve model robustness and cross-target generalizability, standardized reporting practices should include complete SELEX metadata including library composition, number of selection rounds, and selection pressures, negative selection datasets, and high-resolution binding metrics. Additionally, structural validation data, including circular dichroism spectra or high-resolution structural information where available, should be reported alongside sequence data. Therefore, adoption of such standardized frameworks will enable reproducibility across laboratories and facilitate the development of generalizable AI models for aptamer discovery.

### 6.2 Molecular docking and structural modeling for aptamer-target and aptamer-drug interactions

Other computational methods, like molecular docking (MD) and structural modeling, complement AI by predicting how aptamers fold and bind their targets. Aptamers adapt intricate 2D or 3D structures like stem-loops, G-quadruplexes that determine binding. Recent studies illustrate the promising potential of *in silico* structure-guided design (127, 128). Structural modeling can also help guide ApDC design for understanding aptamer-drug interactions and to predict whether the payload will sterically hinder aptamer-target binding or how it will be exposed for release (33). Though only a few studies report the explicit docking of aptamers to free drugs, several MD studies have examined drug-aptamer stability (122, 129–131). For instance, doxorubicin is known to form π-π stacking with DNA aptamers like AS1411, and MD simulations can help understand aptamer folding changes upon drug binding and release (132). In principle, an ApDC computational pipeline first helps model the aptamer-target complex. Subsequently, with the help of MD, it becomes easier to predict whether the conjugate still docks properly. Thus, docking and structural modeling are potent tools for refining aptamer-target interactions and can be extended to ApDCs to ensure the payload does not disrupt binding.

### 6.3 Multi-omic and spatial data integration for aptamer target selection

Target selection for aptamers is based on identifying biomarkers that distinguish tumor cells or immunosuppressive cells from normal cells. Integrative multi-omic analyses have been increasingly used to discover such targets. High-throughput proteomic technologies like SOMAscan can help quantify thousands of proteins in plasma or tissue (133). Furthermore, such data can then be cross-referenced with RNA-seq and metabolomics to validate target overexpression and pathway context (134). In addition, spatial transcriptomics and imaging add a further layer by helping map cell types *in situ* to reveal microenvironment details on where target expression is localized (135). Such spatial maps can thus potentially help guide the design of aptamers to target immune-suppressive niches (136). Recent techniques can now also merge aptamer-binding data with sequencing for single-cell analyses. For instance, an Apt-seq method was developed that works by attaching poly-A tails to aptamers and sequencing them alongside cellular RNA (137). This allows for a simultaneous profiling of cell-surface epitopes via aptamers and also permits simultaneous gene expression in thousands of cells. Thus, this method is promising as it offers a powerful multi-omic readout and aid in correlating aptamer binding intensity to expression of thousands of genes. Therefore, candidate targets can be further validated functionally by confirming aptamer binding based on the predicted cell type. Furthermore, with the integration of ML algorithms like unsupervised clustering or graph-based methods, signature combinations of genes and proteins can be identified that define tumor or immune subsets. Hence, the high multiplexing capacity of aptamers holds promising potential to close the gap between genomics and proteomics.

## 7 Manufacturing and regulatory considerations

### 7.1 GMP synthesis and purification of chemically modified aptamers and ApDCs

Aptamers and ApDCs are commonly developed via solid-phase phosphoramidite chemistry, which is a well-established GMP process (30). As discussed in the previous sections, aptamers are often further chemically modified to improve nuclease resistance and pharmacokinetics. In addition, in ApDCs, drug-loaded phosphoramidite modules are designed such that the cytotoxins or drug moieties with cleavable linkers can be incorporated directly into the oligonucleotide chain at defined positions (138). For manufacturing GMP aptamers and ApDCs, first, an automated solid-phase assembly is carried out during which oligonucleotide chains are built over solid support systems. During this process, the coupling yields are optimized using validated reagents and strict process control (139). In the case of ApDCs, drug conjugation is also carried out using a specialized module bearing the drug and a cleavable linker. Subsequently, the full-length aptamer or the ApDC is cleaved from the solid support and deprotected. The resultant crude product is then finally purified to separate the full-length conjugates from truncated or failed sequences (140).

### 7.2 Quality control and stability testing

The final GMP batch of aptamer or ApDC products also undergoes comprehensive quality control to confirm identity, purity, and stability. High-resolution mass spectrometry is used to verify the identity and composition of the resultant products (141). Other mass analysis methods like liquid chromatography-electrospray ionization-mass spectrometry (142) or matrix-assisted laser desorption mass spectrometry (143) are applied to verify the molecular weight of the full-length aptamer or ApDC. In addition, UV spectroscopy and elemental analysis further aid in confirming the aptamer composition (144). Orthogonal chromatographic methods are used to quantify the purity of the aptamer products (145). Reverse-phase HPLC and ion-exchange HPLC separate full-length product from failure sequences, truncated chains, and chemical impurities (146, 147). Also, because aptamer activity depends on folding, secondary or tertiary structure is assessed using techniques like circular dichroism (CD) or UV melting (148). More importantly, the stability of aptamer and ApDC products must also follow the ICH Q1 guidelines. Thus, forced-degradation studies are performed to map breakdown pathways (140). In addition, other analytical methods, such as liquid chromatography with UV and mass spectrometry detection and ion-exchange high-performance liquid chromatography, are used to detect subtle impurities in aptamers or ApDCs (149, 150).

In addition, for ApDCs, an additional analytical challenge lies in confirming that payload attachment does not disrupt aptamer folding or target binding that requires comparative structural and functional characterization of the aptamer before and after conjugation. Circular dichroism spectroscopy (151) and thermal melting analysis can be used to assess secondary structure integrity (152), while surface plasmon resonance (153) or biolayer interferometry (154) can quantify any changes in binding affinity and kinetics. High-resolution mass spectrometry and size-exclusion chromatography further confirm structural integrity and conjugation efficiency (155). Moreover, complementary molecular dynamics simulations can provide insight into steric hindrance or conformational perturbations induced by the payload or linker (156). Together, these orthogonal approaches can be applied to ensure that functional activity is preserved in the final ApDC construct.

### 7.3 Regulatory challenges and IP and commercialization landscape

The US Food and Drug Administration (FDA) recently issued a guidance on the clinical pharmacology of ADCs that discusses their essentials, like dosing, bioanalysis, and immunogenicity, which may shape the ApDC development and regulation (157). In Europe, the European Medicines Agency (EMA) treats chemically synthesized oligonucleotides as medicinal products and thus advises sponsors to address the legal framework if claiming any new active substance status for a conjugate when an unconjugated counterpart exists (140). Therefore, both FDA and EMA expect full CMC compliance, as for any drug that is manufactured, and control strategies for the aptamer and the payload must each meet drug standards. Furthermore, regulatory authorities also require characterization of the conjugated versus unconjugated forms. EMA notes that for conjugated oligonucleotides, both moieties should be separately analyzed and must be compared with pre- and post-conjugation to confirm that the drug has not disrupted the aptamer folding or binding. Immunogenicity is another factor that needs to be considered. However, additional bridging studies are required to substantiate the existing frameworks for aptamers and ApDCs.

Furthermore, despite significant preclinical progress, several critical gaps continue to limit the clinical translation of ApDCs. First, pharmacokinetic instability and rapid clearance remain major barriers to achieving sustained therapeutic exposure (158). Second, the absence of standardized pipelines linking SELEX-derived candidates to clinical-grade development results in variability in candidate quality and reproducibility (159). Third, compared to antibody-based therapeutics, there is a limited pool of clinically validated aptamer targets with well-characterized expression profiles and internalization kinetics (13). Finally, manufacturing challenges related to large-scale synthesis, folding consistency, and conjugation reproducibility must be addressed to meet regulatory requirements (160). Bridging these gaps will be essential for advancing ApDCs from experimental platforms to clinically viable therapeutics.

In addition, recently, aptamers have become a rising field of IP filing with ~135,000 aptamer-related patents in the past decade alone (161), reflecting the promising applications of aptamers as diagnostics, therapeutics, and molecular tools. A recent 2024 market report estimated that the global aptamer market stands at ~$1.94 billion in 2022 alone, and is growing ~25% annually, primarily driven by diagnostics and drug discovery applications (161). However, despite the growing interest, there are only a few commercialized aptamer drugs currently available. The first FDA-approved aptamer therapeutic is pegaptanib (Macugen), which is a PEGylated VEGF aptamer for wet age-related macular degeneration that was approved in 2004 (162). Also, currently, no ApDC has been commercialized yet. Thus, in oncology immunotherapy, most aptamers work remains preclinical, though researchers are exploring PD-1/PD-L1 and other checkpoint-targeting aptamers and conjugates, as discussed in the previous sections. Moreover, commercialization depends on multiple factors, especially on clearing IP hurdles and securing partnerships. Thus, researchers must also navigate both global patent coverage and freedom-to-operate. Strategic alliances and licensing will likely play a key role as ApDC technology moves from laboratory to clinic.

## 8 Future perspectives

As discussed in the previous sections, the next-generation aptamer-based immunotherapies will increasingly emphasize multi-functional and programmable constructs. Instead of simple one-to-one binders, future designs will integrate multiple targeting and regulatory features into single molecules (163, 164). With further work, tri-specific and multivalent aptamer constructs bearing two or more distinct binding domains can be developed such that a single aptamer can bind to multiple targets or cells (163). In addition, switchable aptamers engineered to change conformation or binding affinity in response to biochemical triggers can also be considered, so that their activity can be modulated based on the tumor microenvironment, thereby allowing a precise temporal control of drug action. Logic-gated aptamer circuits that implement Boolean logic on molecular inputs are another promising application that requires the presence or absence of multiple antigens before activating, thereby drastically enhancing specificity, and can also be considered (165). Dual-checkpoint aptamers designed to target two immune checkpoints at once are another promising area that can be explored so that such a system can effectively relieve immune inhibition on two fronts with a single molecule (6). Therefore, such multi-functional architecture promises to increase on-target potency and reduce side effects. By combining multiple targeting and regulatory functions, next-generation aptamers can allow for complex immunomodulation and thus pave the way for programmable aptamer therapies that operate with antibody-like potency but with the tunability of nucleic acids.

### 8.1 Synergy with cell therapies and mRNA immunotherapies

Aptamers are increasingly being integrated with cutting-edge cancer therapies to enhance efficacy and safety. For adoptive cell therapies like chimeric antigen receptor T-cell (CAR-T), chimeric antigen receptor natural killer cell (CAR-NK), and tumor-infiltrating lymphocytes (TILs), aptamers offer novel targeting and control mechanisms. For instance, aptamers can serve as the antigen-recognition domain of engineered T cells without genetic modification, as seen in a recent study in which researchers replaced the scFv in a CAR construct with a DNA aptamer sequence (79). These Apt-CAR T cells can thus be activated by binding the target antigen via the aptamer and killing tumor cells *in vitro* and *in vivo* (79). In addition, the antigen-binding strength and specificity can be tuned by altering the aptamer, and additional features like sensitivity to tumor-microenvironment ATP levels (79). Thus, such a non-genetic plug-and-play strategy allows for easy retargeting of the same CAR-T platform, especially against new antigens and the integration of switchable binding motifs without re-engineering the T cells.

Furthermore, aptamers can also arm immune effector cells. For instance, in a recent study, NK cells were functionalized with multivalent aptamer complexes, including four copies of a CD30-specific aptamer that were attached to NK cells via click chemistry, providing the NK cells with precise homing to CD30+ lymphoma cells (166). Such aptamer-guided NK cells can bind to tumor cells with more efficiency than unmodified NK cells and thus provide markedly higher cytotoxicity in both cell culture and animal models. In principle, similar methods can also be applied for CAR-NK or ILs, hence potentially granting them new specificity or safety control by an aptamer trigger. In addition, aptamer-based cell sorting and activation techniques are also emerging tools that can be considered, which can streamline the manufacturing of adoptive cell products.

Aptamers can also guide mRNA-delivering nanoparticles to the desired immune or tumor cells. For instance, lipid nanoparticles (LNPs) bearing cell-specific ligands have shown to alter mRNA delivery profiles significantly. In a recent study, an LNP conjugated to a CD4 antibody provided a >30-fold increase in mRNA expression in CD4+ T cells compared to untargeted LNPs (167, 168). Likewise, LNPs attached to an EGFR-targeting aptamer have also shown an enhanced uptake by EGFR+ cancer cells *in vivo* (168). Therefore, aptamer-functionalized LNPs can be potentially used to deliver mRNA vaccines or therapeutic transcripts specifically to dendritic cells or tumor cells. Moreover, aptamer-guided mRNA delivery can also enable *in vivo* engineering of cells. Thus, these strategies bear promising potential for amplification of the potency of mRNA immunotherapies by merging them with the precision targeting capabilities of aptamers (169).

### 8.2 Integration into precision immuno-oncology pipelines

Owing to their small size, chemical synthesis, and ease of modification of aptamers, they are highly adaptable for personalized use. One such example is the aptamer-nanoparticle vaccines that can be customized on demand. By exchanging an aptamer ligand or the encapsulated cargo, the same delivery platform can be redirected to different immune cells or tumor antigens. In personalized mRNA vaccines, aptamers enable cellular precision (169). Similarly, aptamer-conjugated nanoparticle systems provide a very flexible platform for immunotherapy that also has the potential to rapidly incorporate new antigen targets or immune activators.

**Figure 5:**
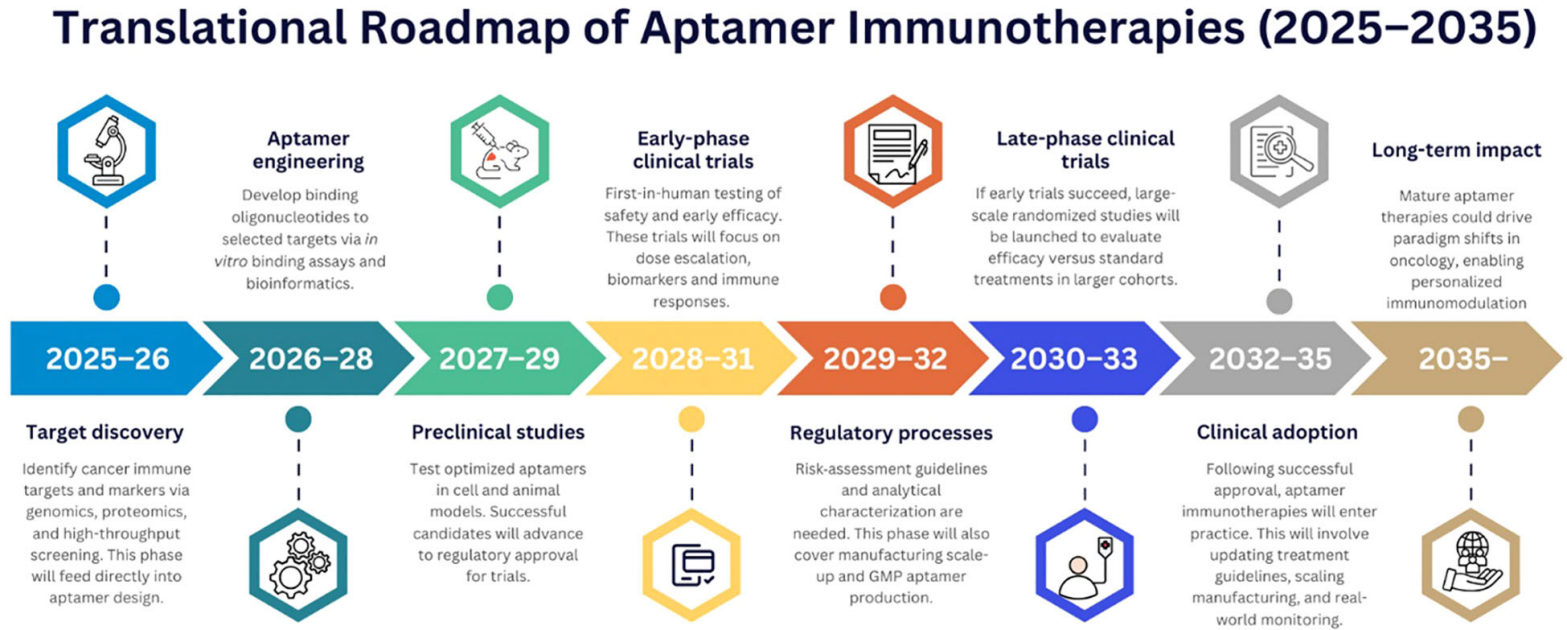
A schematic illustration showing the translational roadmap of aptamer immunotherapies (2025–2035).

Apart from targeted delivery, aptamers can also serve as biomarkers and diagnostics to drive precision decisions (170). High-throughput SELEX can help generate aptamers against patient-specific biomarkers, thereby allowing for truly individualized targeting (171). In addition, aptamer panels could be applied for multiplexed immunophenotyping or liquid biopsy, as has been done with SOMAmer technology in proteomics (172). Moreover, integration with bioinformatics and ML can potentially help automate matching aptamer sequences to the mutational landscape of the tumor. Therefore, aptamers offer a highly programmable toolkit that integrates with genomic and immunologic data, thereby enabling on-demand customization of personalized immunotherapies. A translational roadmap of the aptamer immunotherapies that can be potentially achieved in the next decade has been highlighted in **Figure 5**. The schematic highlights the progressive evolution of aptamer technologies from current preclinical and early clinical investigations toward more advanced, clinically approved therapeutic platforms. In the near term, the roadmap emphasizes the optimization of aptamer design, including improvements in stability, binding affinity, and *in vivo* pharmacokinetics, alongside the expansion of SELEX-derived libraries targeting immune checkpoints and tumor-associated markers. The mid-term phase focuses on translational advancement, with increased entry of aptamer-based candidates into clinical trials. Looking ahead to the long-term horizon, the roadmap envisions the clinical approval and widespread adoption of aptamer immunotherapies as part of standard cancer treatment regimens.

## 9 Conclusions

Aptamers were initially isolated as nucleic acid probes for diagnostic applications and have now also applied as sophisticated therapeutic ligands (10), particularly because of their inherent advantages, including high affinity, structural stability, and extremely low immunogenicity that allows them to be synthesized and modified easily on a large scale (1, 173). These properties enable aptamers to be exploited as programmable immune modulators for clinical therapeutic applications (173). Aptamers have also been engineered as antagonists or agonists of immune checkpoints, thus confirming their superior selectivity, modularity, and lower immunogenicity as compared to monoclonal antibodies (173). Moreover, aptamer-drug conjugates exemplify the integration of targeted therapy and immune modulation. By chemically linking a cytotoxic or immunomodulatory payload to an aptamer targeting domain, ApDCs deliver therapy with antibody-like precision but with chemical-synthesis advantages (107). Furthermore, because aptamers involve nucleic acid components, ApDCs are inherently antibody-free and more easily scalable, and thus large quantities can be manufactured via chemical synthesis without involving any biological steps. This chemical versatility also enables combination therapies, that is, aptamers can be easily conjugated to cytokines, TLR agonists, or siRNAs, enabling simultaneous targeted cytotoxicity and modulation of the tumor immune microenvironment. Hence, ApDCs offer a modular immunotherapy platform that merges precision targeting with programmable payload delivery.

Moving forward, successful clinical applications will require integration of advanced chemistry, computational design, and standardized development pipelines. In addition, novel linker and backbone modifications must be further explored to enhance *in vivo* stability, half-life, and payload control. With improved SELEX protocols and AI-driven modeling integration, aptamer discovery and optimization can be further accelerated. To translate these advances, the field must adopt standardized frameworks for evaluation, such as consensus protocols for aptamer screening and characterization. Initiatives like the proposed MAPS guidelines for aptamer selection must also be applied to remain consistent with the assay standards and reporting (174). More research work must prioritize collaborative efforts to translate synthetic immune modulators into next-generation cancer therapies. Thus, by combining cutting-edge chemistry and computation with rigorous validation, aptamer-based immunotherapies can be scaled safely from research laboratories to clinical applications.
